# Phenolic Acid Composition and In Vitro Antioxidant Capacity in the Flesh of Thirty-Three *Cucurbita pepo* Accessions

**DOI:** 10.3390/foods15122226

**Published:** 2026-06-20

**Authors:** Ying Xiang, Jing Yu, Xuexue Wang, Kaiwen Gu, Jinsong Bao, Xiaoyong Xu

**Affiliations:** 1 College of Agriculture and Biological Engineering, Taizhou Vocational College of Science & Technology, Taizhou 318020, China; xiangying6767@163.com; 2 Institute of Nuclear Agricultural Sciences, College of Agriculture and Biotechnology, Zhejiang University, Hangzhou 310058, China; yjingyx@163.com (J.Y.); 17353655845@163.com (X.W.); 22516184@zju.edu.cn (K.G.); 3 Hainan Institute, Zhejiang University, Yazhou Bay Science and Technology City, Sanya 572025, China; 4 Hainan Seed Industry Laboratory, Yazhou Bay Science and Technology City, Sanya 572025, China

**Keywords:** *Cucurbita pepo*, phenolic acids, antioxidant capacity, germplasm characterization

## Abstract

To characterize intraspecific variation in phenolic acid composition and in vitro antioxidant capacity, color parameters, total phenolic contents (TPC), hydrolyzable phenolic acid profiles, and DPPH and ABTS radical scavenging capacities were systematically determined in the flesh of 33 *Cucurbita pepo* accessions. All accessions exhibited bright yellow flesh, with significant variation in red-green value (a). TPC and antioxidant capacity differed markedly among accessions and generally followed right-skewed distributions, indicating that a limited number of accessions accumulated high levels of phenolics and antioxidant activity. Eight phenolic acids were quantified by high-performance liquid chromatography (HPLC), with *p*-hydroxybenzoic acid (8.97–341.98 μg/g), *p*-coumaric acid (2.42–761.88 μg/g), and ferulic acid identified as the major compounds. Ferulic acid and caffeic acid showed strong positive associations with both DPPH and ABTS scavenging capacities. Hierarchical clustering separated the accessions into two major groups, with Group 2 exhibiting higher TPC (208.89–657.69 µg GAE/g), total phenolic acid content (109.92–890.85 µg/g), and ABTS antioxidant capacity than Group 1. The high-antioxidant accessions may serve as promising candidates for antioxidant-enriched *C. pepo* products and quality-oriented breeding.

## 1. Introduction

Phenolic compounds, a large and diverse class of plant secondary metabolites characterized by one or more phenolic hydroxyl groups, are widely distributed in plant tissues [1]. These compounds are abundant in dietary sources such as fruits, vegetables, and staple crops, and are structurally categorized into phenolic acids, flavonoids, lignans, and tannins [1,2,3]. Among these, phenolic acids are one of the most common subclasses and mainly comprise hydroxybenzoic acid derivatives, such as gallic acid, protocatechuic acid, *p*-hydroxybenzoic, vanillic, and syringic acid, and hydroxycinnamic acid derivatives, such as caffeic, *p*-coumaric, ferulic, sinapic, and chlorogenic acid [3,4]. Increasing evidence indicates that dietary phenolic compounds possess multiple bioactivities, including antioxidant, anti-inflammatory, and antidiabetic activities [3,5]. However, their bioactivity and nutritional value are not only determined solely by the total content, but also strongly influenced by their specific chemical structures, molecular forms, and interactions within complex food matrices [2,6,7]. Therefore, comprehensive profiling of individual phenolic components is essential for accurately assessing the nutritional and functional value of plant-derived foods.

The genus *Cucurbita* comprises several economically and nutritionally vital crops cultivated worldwide, among which *Cucurbita pepo*, *C. moschata*, and *C. maxima* are the three major cultivars [8,9]. As one of the earliest domesticated cucurbit crops, *C. pepo* exhibits extensive phenotypic and genetic diversity and includes commercially important morphotypes such as zucchini and spaghetti squash [9,10]. This species is widely consumed across geographically diverse regions, including Austria, Hungary, Mexico, Slovenia, China, Spain, and several Asian and African countries [11]. The immature fruits are consumed as vegetables, whereas mature fruits are palatable and eaten either raw or processed (e.g., roasted or cooked) in pastries, vegetable dishes, beverages, and baked products [12]. Nutritionally, *C. pepo* fruits are rich sources of proteins, amino acids, dietary fiber, fatty acids, minerals, and vitamins, with considerable variation across genotypes and tissues [11,13].

In addition to primary nutritional value, *C. pepo* fruit quality is strongly influenced by bioactive phytochemicals, particularly phenolic compounds and carotenoids [11,14]. Carotenoids, including β-carotene, lutein, and related pigments, are major determinants of yellow to orange flesh color in pumpkin and may also contribute to antioxidant properties [13,14]. Recent studies have identified diverse phenolic constituents (e.g., caffeic acid, *p*-hydroxybenzoic acid, *p*-coumaric acid, vanillic acid, and gallic acid) in different *Cucurbita* tissues, including flesh, peel, and seeds [12,14]. These metabolites have been associated with in vitro antidiabetic activities such as the inhibition of α-glucosidase [12]. Moreover, the fruit flesh, which is the primary edible portion, has exhibited remarkable bioactivity, with reported DPPH radical scavenging activities exceeding 76% in certain genotypes [15]. These findings suggest that *C. pepo* flesh may represent a valuable source of antioxidant-related phytochemicals.

Despite these advances, several knowledge gaps remain. First, many previous studies have focused on total phenolic content or broad phytochemical screening rather than the targeted quantification of individual phenolic acids [16,17]. Second, characterization of phenolics in *Cucurbita* species has often emphasized non-flesh tissues or by-products, such as peels, seeds, and flowers, whereas the edible flesh has received comparatively less attention [8,14,18]. Third, although color is an important quality trait of pumpkin flesh, the relationships among color parameters, phenolic acid composition, and antioxidant capacity remain insufficiently clarified.

In this study, 33 *Cucurbita pepo* accessions cultivated under uniform conditions were analyzed to systematically characterize phenolic acid composition and antioxidant capacity in the fruit flesh. Individual phenolic acid profiles were quantified, and in vitro antioxidant capacities were evaluated using DPPH and ABTS assays. Multivariate statistical analyses were further employed to explore the relationships among color parameters, phenolic traits, and antioxidant capacity, as well as to preliminarily distinguish accessions showing different phytochemical and antioxidant profiles. This study advances current knowledge by providing a targeted assessment of accession-dependent variation in phenolic acid composition in the edible flesh of *C. pepo*, thereby offering useful information for germplasm characterization and future phytochemical quality improvement.

## 2. Materials and Methods

### 2.1. Plant Materials

A total of thirty-three *Cucurbita pepo* accessions representing diverse genetic backgrounds were used in this study. For clarity, these accessions were designated as Sample 1 to Sample 33 (S1–S33). All accessions were cultivated in an experimental field in Yuncheng City, Shanxi Province, China, during early April to late July 2024. Fruits were harvested in late July 2024, corresponding to 60–66 days after flowering. After harvest, the peel and seeds were removed to isolate the edible flesh. The flesh samples were vacuum freeze-dried (SCIENTZ-12N/A, Scientz Freeze-Drying, Ningbo, China), ground into homogeneous powder, and stored at 0–4 °C in the dark until phenolic extraction and compositional analysis.

### 2.2. Chemicals

ABTS [2,2′-azino-bis(3-ethylbenzothiazoline-6-sulfonic acid) diammonium salt], DPPH (2,2-diphenyl-1-picrylhydrazyl), Folin–Ciocalteu reagent, and Trolox (6-hydroxy-2,5,7,8-tetramethylchromane-2-carboxylic acid) were purchased from Sigma-Aldrich Chemical Co. (St. Louis, MO, USA). The standards of gallic acid (GA), protocatechuic acid (PA), *p*-hydroxybenzoic acid (*p*-HA), vanillic acid (VA), caffeic acid (CaA), sinapic acid (SA), *p*-coumaric acid (*p*-CA), and ferulic acid (FA) were also obtained from Sigma-Aldrich Chemical Co. (St. Louis, MO, USA). Hydrochloric acid, potassium persulfate and sodium hydroxide were purchased from Sinopharm Chemical Reagent Co., Ltd. (Shanghai, China). HPLC grade methanol and ethyl acetate were purchased from Merck (Darmstadt, Germany) and Tedia (Fairfield, OH, USA), respectively. HPLC-grade acetic was sourced from Macklin (Shanghai, China).

### 2.3. Color Parameters

The color parameters of freeze-dried flesh powder were measured using a spectrophotometer (NS800, Shenzhen ThreeNH Technology Co., Ltd., Shenzhen, China), including lightness (L), red-green (a) and yellow-blue value (b). Calibration was performed using a standard white plate before measurement.

### 2.4. Extraction of Phenolic Compounds

Phenolic compounds were extracted according to Yu et al. [19], with minor modifications. Briefly, 0.5 g of freeze-dried flesh powder was extracted twice with 10 mL of 80% methanol for 30 min using a multi-speed oscillator (HY-8, Changzhou Guohua Electric Appliance Co., Ltd., Changzhou, China). After each extraction, the mixture was centrifuged at 9400× *g* for 10 min at 4 °C, and the supernatants were combined and concentrated at 37 °C under vacuum using a rotary evaporator (RE-2000A, Ya Rong Biochemistry Instrument Factory, Shanghai, China). The concentrated extracts were hydrolyzed with 4 M NaOH for 2 h to release conjugated and ester-bound phenolic acids, acidified to pH 1.5–2.0, and extracted with ethyl acetate. The ethyl acetate extracts were evaporated and reconstituted in 3 mL of 50% methanol. All extractions were performed in triplicate, and the extracts were stored at −20 °C in the dark until analysis.

### 2.5. Determination of Total Phenolic Content

Total phenolic content (TPC) was determined using the Folin–Ciocalteu assay as described by Shao et al. [20]. Briefly, 0.2 mL of the phenolic extract was mixed with 1.5 mL of 0.1 N Folin–Ciocalteu reagent, followed by the addition of 1.5 mL of saturated sodium carbonate solution. After incubation in the dark for 90 min, the absorbance was measured at 725 nm using an Infinite M Nano microplate reader (Tecan Group Ltd., Männedorf, Switzerland). Gallic acid was used for calibration, and the results were expressed as micrograms of gallic acid equivalents per gram of freeze-dried flesh powder on a dry weight basis (μg GAE/g). All measurements were performed in duplicate.

### 2.6. HPLC Analysis of Phenolic Acids

**Figure 1:**
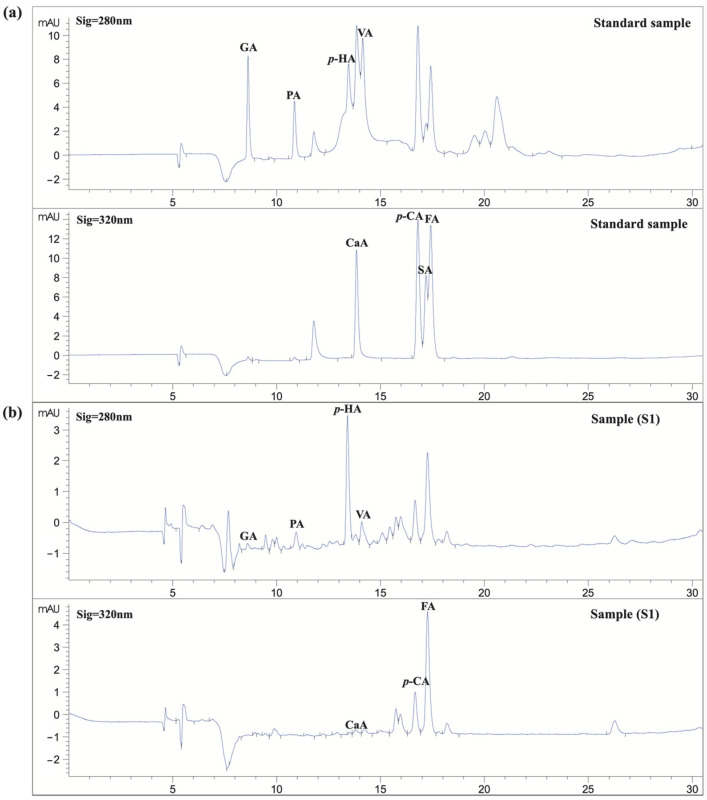
HPLC chromatograms of phenolic acids. (**a**) The standards of phenolic acids detected at 280 and 320 nm; (**b**) Phenolic acids of samples detected at 280 and 320 nm. GA, gallic acid; PA, protocatechuic acid; *p*-HA, *p*-hydroxybenzoic acid; VA, vanillic acid; CaA, caffeic acid; *p*-CA, *p*-coumaric acid; SA, sinapic acid; FA, ferulic acid. The same abbreviations apply below.

Phenolic acids, mainly hydrolyzable phenolic acids, were analyzed by an HPLC system (Agilent Technologies 1260 Infinity, Santa Clara, CA, USA), according to the methods described by Yu et al. [19]. Before analysis, all extracts were filtered through 0.45 μm membrane filters. Separation was performed on an Agilent Eclipse Plus C18 column (250 mm × 4.6 mm, 5 μm; Agilent Technologies, Santa Clara, CA, USA) maintained at 35 °C. The mobile phase consisted of 0.1% acetic acid in water (A) and 0.1% acetic acid in methanol (B). The flow rate was 0.5 mL/min, and the injection volume was 3 μL. A 35 min linear gradient was applied as follows: 0–1 min, 9–25% B; 1–4 min, 25–35% B; 4–8 min, 35–45% B; 8–10 min, 45–46% B; 10–12 min, 46–47% B; 12–14 min, 47–48% B; 14–16 min, 48–48.5% B; 16–16.5 min, 48.5–49% B; 16.5–17.5 min, 49–49.1% B; 17.5–18 min, 49.1–49.5% B; 18–20 min, 49.5–50% B; 20–25 min, 50–60% B; 25–30 min, 60–9% B; 30–35 min, 9% B. All phenolic acids were quantified using external calibration curves based on the retention time of authentic standards (Figure 1). The results were expressed as micrograms per gram of freeze-dried flesh powder on a dry weight basis (μg/g). Total phenolic acid content (TPA) was calculated as the sum of all quantified phenolic acid fractions. Each extract was analyzed in duplicate. The regression equations, limit of quantification (LOQ) and recovery rate data are provided in Table S1. For accessions with phenolic acid content below the LOQ, the extract was concentrated and re-analyzed to enable detection.

### 2.7. Determination of Antioxidant Capacity

In vitro antioxidant capacity was measured using ABTS and DPPH radical scavenging assays according to the method reported by Pang et al. [21]. The phenolic extracts were used for both assays, and the results were expressed as micromoles of Trolox equivalents per gram of freeze-dried flesh powder on a dry weight basis (μM TE/g). Each extract was measured in duplicate.

### 2.8. Statistical Analysis

All data are presented as means ± standard deviation (SD). Analysis of variance (ANOVA) was performed with SAS software version 8.0 (SAS Institute Inc., Cary, NC, USA), and mean comparisons were conducted using Tukey’s test at *p* < 0.05. Pearson correlation analysis was performed at significance levels of *p* < 0.05 and *p* < 0.01. Principal component analysis (PCA) and hierarchical cluster analysis were conducted using Origin software 2021 (OriginLab Corporation, Northampton, MA, USA). Prior to cluster analysis, all variables were standardized using Z-score normalization. Hierarchical clustering was performed using Euclidean distance and Ward’s linkage method. Box plots were generated using GraphPad Prism 10.0 (GraphPad Software, Boston, MA, USA).

## 3. Results

### 3.1. Color Parameters, Total Phenolic Content, and Antioxidant Capacity

Substantial variation in color parameters, TPC, and antioxidant capacity was observed among the 33 *C. pepo* accessions (Table 1). Color measurements showed that all accessions had bright yellow flesh. The lightness value (L) ranged from 75.72 to 85.64, with a mean value of 82.46, indicating relatively limited variation in flesh brightness. The yellow-blue value (b) ranged from 19.18 to 35.22, with a coefficient of variation (CV) of 15.72%, indicating moderate variation in yellow coloration. In contrast, the red-green value (a) showed greater variability, with a CV of 56.29%, indicating pronounced differences in red–green color attributes.

TPC varied considerably among accessions, ranging from 113.06 to 657.69 μg GAE/g. The highest TPC were observed in S21, whereas the lowest values were detected in S1, S2 and S3 (Table 1). The relatively high CV for TPC (42.65%) indicated considerable accession-dependent variation in phenolic accumulation. The DPPH and ABTS assays also revealed notable differences in antioxidant capacity, with CV values of 25.28% and 31.17%, respectively. For DPPH radical scavenging capacity, the highest values were observed in S4, S16, and S17, whereas the lowest value was detected in S2, with an overall mean of 0.65 μM TE/g. Similarly, ABTS radical scavenging capacity peaked in S20, S4, and S22, and lowest in S1 and S2, with a mean value of 1.17 μM TE/g. Overall, most parameters showed positive skewness, indicating a generally right-skewed distribution characterized by many low-to-moderate values and a few high-value accessions (Table 1).

### 3.2. Profiling of Individual Phenolic Acids

Because alkaline hydrolysis was applied during extraction, the individual phenolic acids quantified in this study mainly represent hydrolyzable phenolic acids. Eight individual phenolic acids were detected across the 33 *C. pepo* accessions (Figure 1b, Table 2), including *p*-HA, *p*-CA, FA, PA, VA, CaA, GA, and SA. Among them, *p*-HA, *p*-CA, and FA constituted the predominant phenolic acid components in pumpkin flesh, whereas the remaining compounds were present at relatively low levels or were undetectable in most accessions. Marked accession-dependent variation was observed in the major phenolic acids. The content of *p*-CA varied widely from 2.42 to 761.88 µg/g, with a median value of 13.59 µg/g, indicating a highly right-skewed distribution characterized by many low-accumulation accessions and a few high-accumulation accessions. Only five accessions, namely S9, S20, S21, S22, and S25, showed *p*-CA levels exceeding 350 µg/g. Similarly, *p*-HA ranged from 8.97 to 341.98 µg/g. Six accessions, including S5, S28, S30, S31, S32, and S33, accumulated more than 200 µg/g *p*-HA. FA was detected in most accessions and reached its maximum level in S4 (65.33 μg/g), whereas it was not detected in S5 and S9. Based on the dominant phenolic acid in each accession, *p*-HA was the major component in 23 accessions, *p*-CA was predominant in nine accessions, and FA was the primary phenolic acid only in S11.

PA and VA were detected as minor phenolic acids, with maximum concentrations of 21.65 and 11.21 µg/g, respectively. CaA, GA, and SA were generally present at trace levels (Tr) or were not detected (ND) in most accessions. TPA ranged from 32.49 µg/g in S3 to 890.85 µg/g in S21, with a high CV of 82.70%. These results indicate substantial heterogeneity in hydrolyzable phenolic acid accumulation among *C. pepo* accessions and are broadly consistent with the variation observed in TPC.

### 3.3. Cluster Analysis

**Figure 2:**
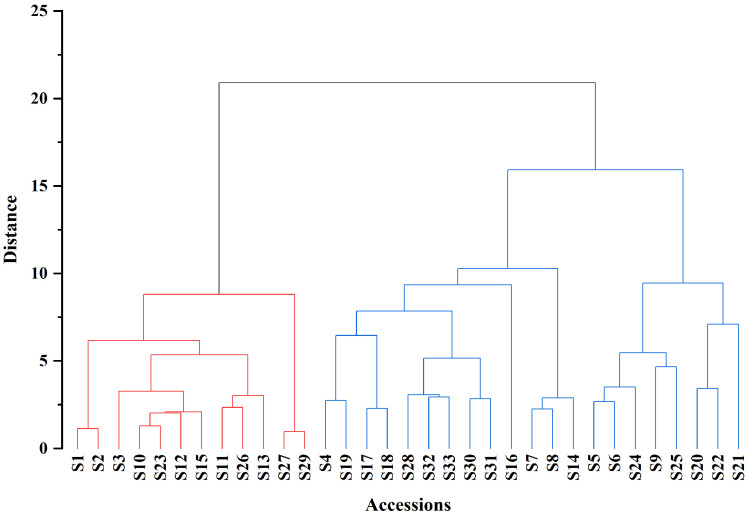
Hierarchical cluster analysis for thirty-three *Cucurbita pepo* accessions. The red branch corresponds to Group 1, and the blue branch to Group 2.

Hierarchical cluster analysis was performed based on color parameters, phenolic acid profiles, TPC, and antioxidant capacity to evaluate the overall similarity among the 33 *C. pepo* accessions (Figure 2). The accessions were divided into two major clusters. Group 1, shown as the red branch, contained 12 accessions, including S1, S10, and S3. These accessions clustered at relatively short linkage distances, suggesting a higher degree of similarity in their integrated quality traits. Group 2, shown as the blue branch, comprised 21 accessions. Compared with Group 1, Group 2 showed longer branch lengths and clustered at higher linkage distances, indicating greater variation in the measured traits within this group. Overall, the clustering pattern indicated marked differences among accessions in their combined color, phenolic, and antioxidant profiles, providing a basis for subsequent group comparison.

### 3.4. Principal Component Analysis

**Figure 3:**
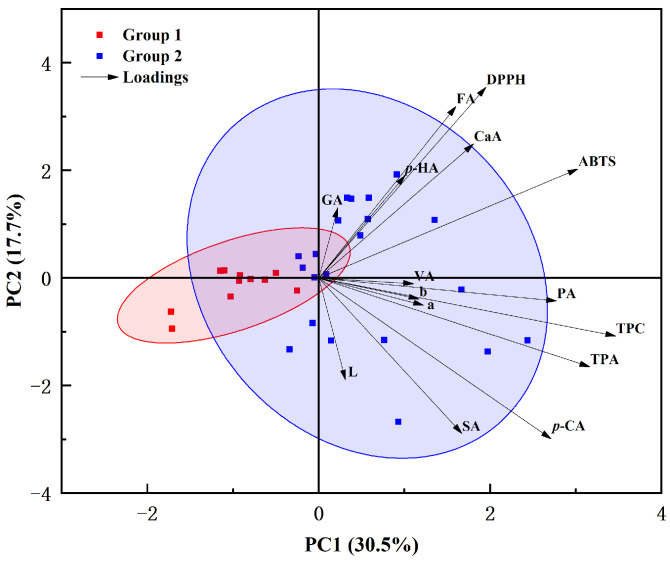
Principal component analysis of color parameters, phenolic compounds, and antioxidant capacity in thirty-three *Cucurbita pepo* accessions. The red and blue circles represent the 95% confidence intervals for Group 1 and Group 2, respectively.

Principal component analysis (PCA) was performed to visualize the distribution patterns of all accessions and to explore the relationships among color parameters, phenolic traits, and antioxidant capacity (Figure 3). The first two principal components explained 48.2% of the total variance, with PC1 and PC2 accounting for 30.5% and 17.7%, respectively. Therefore, PC1 and PC2 captured the major but not complete variation among accessions. Notably, the first six principal components cumulatively explained 83.7% of the total variance, indicating that subsequent components also contributed to the variation (Table S2).

The two groups identified by hierarchical clustering showed partial separation along the PC1 axis, with most Group 2 accessions located on the positive side of PC1. The loading plot indicated that PC1 was mainly associated with TPC, TPA, and ABTS radical scavenging capacity (Table S2), suggesting that phenolic accumulation and ABTS antioxidant capacity contributed substantially to group separation. In contrast, PC2 was more closely related to DPPH radical scavenging capacity and ferulic acid (FA), indicating that these variables accounted for additional variation among accessions, particularly within Group 2. The main variables contributing to PC3, PC4, PC5, and PC6 were b color parameters, *p*-HA, GA, and VA, respectively, suggesting that the remaining variation was associated with differences in color traits and individual phenolic acids (Table S2).

### 3.5. Correlation Analysis

**Figure 4:**
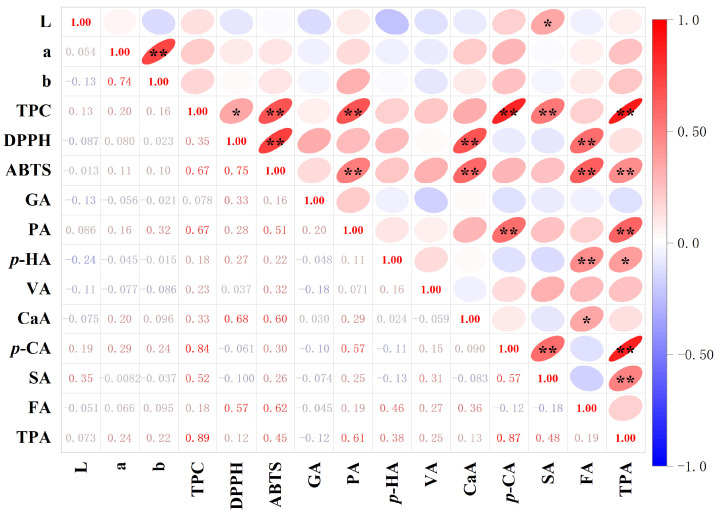
Correlation analysis of color parameters, phenolic compounds, and antioxidant capacity. * and ** indicate significances at *p* < 0.05 and *p* < 0.01, respectively.

Pearson correlation analysis was performed to examine the relationships among color parameters, phenolic traits, and antioxidant capacity (Figure 4). ABTS radical scavenging capacity exhibited a strong positive correlation with both TPC (*r* = 0.67, *p* < 0.01) and TPA (*r* = 0.45, *p* < 0.01), while DPPH radical scavenging capacity showed a weaker but significant correlation with TPC (*r* = 0.35, *p* < 0.05). Among individual phenolic acids, FA (*r* = 0.57–0.62, *p* < 0.01) and CaA (*r* = 0.60–0.68, *p* < 0.01) showed relatively strong positive correlations with antioxidant capacity, suggesting that these compounds may be associated with the observed antioxidant variation among accessions.

Significant correlations were also observed among individual phenolic acids. For example, *p*-CA was positively correlated with PA and SA, while FA showed a positive association with *p*-HA (*p* < 0.05). These associations may reflect coordinated variation in phenolic acid accumulation among accessions. In contrast, color parameters showed no significant correlations with the measured phenolic acid variables, except for a strong positive correlation between a and b values (r = 0.74, *p* < 0.01). Overall, the correlation results indicate that antioxidant capacity was more closely associated with phenolic traits than with color parameters.

### 3.6. Comparative Analysis of Phenolic Content and Antioxidant Capacity Between Groups

**Figure 5:**
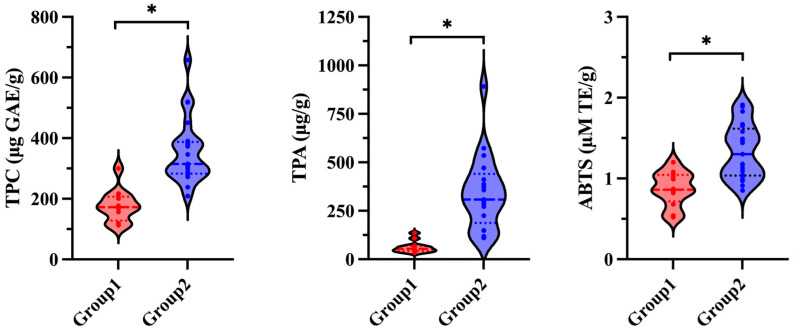
Comparative analysis of total phenolic content, phenolic acids and antioxidant capacity between two groups. The dashed lines indicate the median and quartiles. A single asterisk (*) indicates a significant difference between the means of the two groups (*p* < 0.05).

Based on the hierarchical clustering and PCA results, TPC, TPA, and ABTS radical scavenging capacity were selected for further comparison between the two groups (Figure 5). In Group 1, TPC and TPA ranged from 113.06 to 300.56 μg GAE/g and from 32.49 to 135.51 μg/g, respectively. In contrast, Group 2 showed higher ranges of TPC and TPA, varying from 208.89 to 657.69 μg GAE/g and 109.92 to 890.85 μg/g, respectively. ABTS radical scavenging capacity was also significantly higher in Group 2 than in Group 1 (*p* < 0.05). Overall, significant differences in TPC, TPA, and ABTS radical scavenging capacity were observed between the two groups (*p* < 0.05). These results indicate that Group 2 represents a high-phenolic group with relatively greater in vitro antioxidant capacity, whereas Group 1 is characterized by comparatively lower phenolic accumulation and antioxidant capacity.

## 4. Discussion

The analysis of 33 *C. pepo* accessions revealed considerable variation in flesh color parameters, phenolic accumulation, and in vitro antioxidant capacity. Among the color parameters, L and b showed limited to moderate variation, whereas a exhibited the highest coefficient of variation, indicating greater accession-dependent differences in red–green coloration. Because color parameters were not significantly correlated with phenolic acid variables (Figure 4), the observed color variation is unlikely to be explained primarily by the measured phenolic acids. Carotenoids are major determinants of yellow-orange pigmentation in *C. pepo* fruits [22]. However, because carotenoids were not quantified in the present study, the variation in color parameters cannot be directly attributed to specific pigment profiles. The significant positive correlation between a and b values may reflects a synchronized transition during ripening. Chlorophyll degradation unmasks the underlying color, while concurrent carotenoid biosynthesis drives the flesh towards yellow and red [23].

Substantial accession-dependent variation was observed in TPC, hydrolyzable phenolic acids, and antioxidant capacity. Because all accessions were cultivated under the same field conditions, the observed differences are likely associated largely with genetic or accession-specific variation. A previous study noted that intraspecific variation in phenolic accumulation often exceeds interspecific differences between *C. pepo* and *C. maxima* [17], highlighting the importance of germplasm evaluation for identifying accessions with desirable phytochemical profiles. The generally right-skewed distributions of TPC, TPA, and antioxidant capacity suggest that most accessions accumulated low-to-moderate levels of phenolics, whereas only a small subset showed relatively high accumulation. This pattern indicates the presence of potentially valuable germplasm for improving phenolic-related quality traits. Although phenolic accumulation is largely under genetic control, previous evidence linking phenylpropanoid metabolism to plant–environment interactions and stress-adaptation pathways [24] suggests that environmental factors may also modulate phenolic traits, warranting multi-location and multi-year validation.

The TPC values observed in this study were lower than those reported in some previous studies on *Cucurbita* species [14,17]. Kulczyński et al. [17] reported that the free TPC in the flesh of *C. pepo* cultivars ranged from 47.2 to 140.4 mg GAE/100 g, *C. moschata* cultivars ranged from 46.6 to 122.0 mg GAE/100 g. Another study reported higher free TPC values of 34.2 to 47.3 mg GAE/g in pumpkin samples [14]. Such differences may be associated with genotype, maturity stage, cultivation environment, and extraction conditions. In particular, the present samples were collected at physiological maturity, whereas younger fruits or different tissues may contain higher levels of phenolic compounds and antioxidant activity [25]. Extraction conditions may also contribute to the observed differences, as aqueous extracts have been reported to yield higher TPC values than aqueous–methanol extracts in some *Cucurbita* samples [17].

The targeted HPLC analysis showed that *p*-HA, *p*-CA, and FA were the predominant hydrolyzable phenolic acids in *C. pepo* flesh. The dominance of these compounds is generally consistent with previous reports on phenolic acid profiles in different *Cucurbita* tissues [14,18,26]. Notably, different accessions exhibited distinct dominant phenolic acid patterns, with most accessions accumulating *p*-HA as the major phenolic acid, whereas several accessions were characterized by high *p*-CA accumulation. Because *p*-coumaric acid is an important intermediate in the phenylpropanoid pathway and can be further converted into caffeic, ferulic, and sinapic acid derivatives [27], the positive correlations observed among *p*-coumaric acid, protocatechuic acid and sinapic acid may indicate coordinated variation in phenolic acid accumulation among accessions. Nevertheless, the underlying biochemical regulation requires further investigation.

The antioxidant capacity of the phenolic extracts was positively associated with TPC and TPA, particularly in the ABTS assay. This finding is consistent with the general view that phenolic compounds contribute to the antioxidant properties of plant-derived foods [17,19]. Recent studies on pumpkin phytochemicals have also shown that phenolic compounds, together with carotenoids and other reducing constituents, contribute to the antioxidant potential (e.g., DPPH and hydroxyl radical scavenging assay) of pumpkin flesh and related tissues [14,25,26]. Among individual phenolic acids, ferulic acid and caffeic acid showed relatively strong positive correlations with antioxidant capacity (*p* < 0.01). These associations may be partly explained by their hydroxycinnamic acid structures and substitution patterns can influence radical scavenging efficiency [4,28]. For example, the methoxy group in ferulic acid may enhance electron delocalization and radical stabilization, while the catechol structure of caffeic acid, characterized by two adjacent hydroxyl groups, may contribute to its radical scavenging activity [29,30]. In contrast, *p*-coumaric acid, which contains only one phenolic hydroxyl group, may exhibit comparatively weaker direct radical scavenging activity. However, it should be noted that these correlations are only statistical associations and do not necessarily imply a direct causal relationship. Moreover, antioxidant capacity in *C. pepo* flesh is likely determined by multiple bioactive constituents, including phenolic acids, flavonoids, carotenoids, tocopherols, vitamin C, and other reducing compounds [25,26]. Therefore, further studies involving compound purification, fractionation, and functional validation are needed to clarify the specific contribution of individual phenolic acids to antioxidant activity.

Multivariate analyses provided an integrated overview of variation among the 33 accessions. Hierarchical clustering separated the accessions into two major groups, and PCA further indicated that phenolic-related traits, particularly TPC, TPA, and ABTS radical scavenging capacity, contributed substantially to group differentiation. Group 2 showed higher levels of TPC, TPA, and ABTS antioxidant capacity than Group 1, suggesting that this group contains accessions with relatively enhanced phenolic accumulation and in vitro antioxidant capacity. With a mean total phenolic content of 356.44 μg GAE/g, Group 2 significantly outperformed non-pigmented polished rice (170–234 μg GAE/g) [19]. Therefore, Group 2 can be considered a promising group for further evaluation. Future studies integrating broader phytochemical profiling, carotenoid determination, multi-environment trials, and biological validation will be necessary to fully assess the functional value and breeding potential of these accessions.

## 5. Conclusions

This study demonstrated substantial accession-dependent variation in flesh color, total phenolic content, hydrolyzable phenolic acid composition, and in vitro antioxidant capacity among 33 *Cucurbita pepo* accessions. *p*-Hydroxybenzoic acid, *p*-coumaric acid, and ferulic acid were the predominant phenolic acids in the flesh. Ferulic acid and caffeic acid were positively associated with antioxidant capacity. Multivariate analyses identified Group 2 as a promising high-phenolic group with relatively strong ABTS radical scavenging capacity. These findings provide useful information for germplasm characterization and for the selection of candidate accessions for future phytochemical quality improvement. Further comprehensive profiling, carotenoid analysis, multi-environment validation, and biological evaluation are warranted.

## Figures and Tables

**Table 1:** Color parameters, total phenolic content and in vitro antioxidant capacity in *Cucurbita pepo* accessions ^1^.

	Color ^2^			Phenolics ^3^		
Accessions	L	a	b	TPC (μg GAE/g)	DPPH (µM TE/g)	ABTS (µM TE/g)
S1	82.7 ± 0.18 ^a–g^	1.98 ± 0.06 ^g–k^	23.41 ± 0.35 ^f–l^	118.8 ± 2.13 ^p^	0.382 ± 0.028 ^jk^	0.539 ± 0.001 ^k^
S2	83.7 ± 0.01 ^a–e^	2.47 ± 0.03 ^e–j^	21.88 ± 1.01 ^i–l^	113.06 ± 8.8 ^p^	0.282 ± 0.024 ^k^	0.52 ± 0.043 ^k^
S3	80.63 ± 0.46 ^e–i^	2.4 ± 0.11 ^f–j^	28.79 ± 0.51 ^b–f^	113.24 ± 0.09 ^p^	0.532 ± 0.008 ^f–k^	0.868 ± 0.019 ^ijk^
S4	85.27 ± 0.11 ^abc^	3.45 ± 0.19 ^c–g^	28.32 ± 0.01 ^b–g^	372.96 ± 2.78 ^d–g^	0.951 ± 0.006 ^abc^	1.888 ± 0.062 ^ab^
S5	85.05 ± 0 ^a–d^	2.28 ± 0.05 ^f–j^	19.57 ± 0.64 ^kl^	381.85 ± 4.63 ^c–f^	0.669 ± 0.061 ^b–j^	0.991 ± 0.094 ^hij^
S6	84.35 ± 0.07 ^a–d^	3.23 ± 0.31 ^c–h^	22.17 ± 0.11 ^i–l^	280.56 ± 21.3 ^h–k^	0.474 ± 0.053 ^h–k^	0.975 ± 0.133 ^hij^
S7	81.74 ± 0.53 ^d–h^	6.67 ± 0.19 ^ab^	33.39 ± 0.58 ^ab^	238.52 ± 11.11 ^j–n^	0.781 ± 0.108 ^a–g^	1.166 ± 0.018 ^f–i^
S8	80.58 ± 1.45 ^e–i^	7.14 ± 0.52 ^a^	35.22 ± 1.66 ^a^	208.89 ± 6.85 ^l–o^	0.557 ± 0.045 ^e–k^	1.184 ± 0.048 ^e–i^
S9	85 ± 0.2 ^a–d^	2.59 ± 0.01 ^d–i^	28.97 ± 0.02 ^b–f^	450.83 ± 17.5 ^bc^	0.478 ± 0.057 ^h–k^	0.854 ± 0.095 ^ijk^
S10	83.49 ± 0.23 ^a–f^	3.86 ± 0.05 ^c–f^	21.64 ± 0.44 ^jkl^	170.65 ± 1.2 ^nop^	0.663 ± 0.046 ^c–j^	0.836 ± 0.077 ^ijk^
S11	82.83 ± 0.64 ^a–g^	3.58 ± 0.14 ^c–g^	27.38 ± 0.57 ^c–i^	156.11 ± 0.93 ^op^	0.634 ± 0.064 ^d–j^	1.084 ± 0.115 ^f–j^
S12	84.32 ± 0.16 ^a–d^	4.95 ± 0.31 ^bc^	26.48 ± 0.16 ^d–j^	173.98 ± 5.28 ^nop^	0.572 ± 0.029 ^d–k^	0.683 ± 0.081 ^jk^
S13	85.64 ± 0.57 ^a^	3.11 ± 0.06 ^d–h^	19.18 ± 0.6 ^l^	167.5 ± 10.65 ^nop^	0.672 ± 0.06 ^b–j^	0.819 ± 0.108 ^ijk^
S14	82.62 ± 0.09 ^a–g^	6.67 ± 0.01 ^ab^	31.64 ± 0.87 ^a–d^	274.35 ± 0.83 ^i–l^	0.706 ± 0.021 ^b–i^	1.025 ± 0.083 ^hij^
S15	82.86 ± 0.23 ^a–g^	4.17 ± 0.76 ^cde^	25.98 ± 2.84 ^e–j^	300.56 ± 20.56 ^hij^	0.587 ± 0.071 ^d–j^	1.047 ± 0.101 ^g–j^
S16	80.91 ± 0.06 ^e–i^	2.68 ± 0.09 ^d–i^	24.86 ± 0.69 ^e–k^	346.02 ± 15.65 ^d–h^	0.958 ± 0.106 ^ab^	1.488 ± 0.128 ^b–f^
S17	82.19 ± 0.18 ^b–h^	0.79 ± 0.08 ^jkl^	22.45 ± 0.02 ^h–l^	380.19 ± 3.33 ^c–f^	1.038 ± 0.096 ^a^	1.647 ± 0.016 ^a–d^
S18	83.25 ± 0.91 ^a–g^	1.61 ± 0.15 ^h–k^	22.2 ± 1.58 ^i–l^	315.28 ± 5.09 ^e–i^	0.794 ± 0.095 ^a–f^	1.46 ± 0.124 ^c–f^
S19	82.9 ± 0.3 ^a–g^	4.96 ± 0.04 ^bc^	29.61 ± 0.22 ^a–e^	310.83 ± 27.69 ^f–i^	0.757 ± 0.058 ^a–h^	1.473 ± 0.051 ^c–f^
S20	83.87 ± 0.63 ^a–e^	3.72 ± 0.08 ^c–g^	24.83 ± 0.19 ^e–l^	518.43 ± 27.13 ^b^	0.707 ± 0.018 ^b–h^	1.912 ± 0.067 ^a^
S21	80.03 ± 1.05 ^g–j^	7.47 ± 1 ^a^	32.89 ± 1.35 ^abc^	657.69 ± 6.57 ^a^	0.669 ± 0.008 ^b–j^	1.44 ± 0.023 ^c–g^
S22	83.11 ± 0.87 ^a–g^	3.09 ± 0.1 ^d–h^	21.81 ± 1.19 ^i–l^	519.91 ± 9.17 ^b^	0.761 ± 0.099 ^a–h^	1.834 ± 0.015 ^abc^
S23	81.97 ± 0.96 ^c–h^	3.76 ± 0.21 ^c–f^	22.79 ± 0.41 ^g–l^	208.8 ± 0.28 ^l–o^	0.603 ± 0.013 ^d–j^	1.019 ± 0.112 ^hij^
S24	85.42 ± 0.07 ^ab^	2.59 ± 0.03 ^d–i^	24.56 ± 0.07 ^e–l^	384.91 ± 21.2 ^cde^	0.416 ± 0.024 ^ijk^	1.297 ± 0.029 ^d–h^
S25	83.19 ± 0.55 ^a–g^	3.56 ± 0.03 ^c–g^	26.58 ± 0.56 ^d–j^	390 ± 4.44 ^cd^	0.499 ± 0.054 ^g–k^	1.035 ± 0.127 ^g–j^
S26	84.36 ± 0.1 ^a–d^	0.77 ± 0.11 ^jkl^	23.82 ± 0.23 ^f–l^	217.13 ± 18.61 ^k–o^	0.612 ± 0.076 ^d–j^	1.196 ± 0.142 ^e–i^
S27	76.72 ± 0.66 ^j^	0.48 ± 0.22 ^kl^	22.84 ± 0.8 ^g–l^	176.94 ± 15.83 ^nop^	0.53 ± 0.051 ^f–k^	0.993 ± 0.099 ^hij^
S28	80.35 ± 0.77 ^f–i^	−0.17 ± 0.04 ^l^	23.37 ± 0.42 ^f–l^	272.59 ± 5.19 ^i–m^	0.557 ± 0.032 ^e–k^	0.91 ± 0.112 ^h–k^
S29	77.71 ± 0.13 ^ij^	1.02 ± 0.09 ^i–l^	21.24 ± 1.16 ^jkl^	201.02 ± 10.09 ^mno^	0.59 ± 0.026 ^d–j^	0.852 ± 0.052 ^ijk^
S30	81.82 ± 0.93 ^d–h^	1.99 ± 0.57 ^g–k^	21.29 ± 1.58 ^jkl^	300.19 ± 0.93 ^hij^	0.835 ± 0.039 ^a–e^	1.579 ± 0.029 ^a–e^
S31	79.07 ± 0.05 ^hij^	4.26 ± 0.44 ^cd^	23.72 ± 0.39 ^f–l^	306.76 ± 0.28 ^g–j^	0.853 ± 0.011 ^a–d^	1.667 ± 0.089 ^a–d^
S32	81.82 ± 0.12 ^d–h^	3.63 ± 0.07 ^c–g^	28 ± 0.22 ^b–h^	285.37 ± 7.59 ^h–k^	0.637 ± 0.076 ^d–j^	1.128 ± 0.051 ^f–i^
S33	81.88 ± 0.48 ^d–h^	2.95 ± 0.47 ^d–h^	25.17 ± 1.87 ^e–k^	289.07 ± 3.7 ^hij^	0.684 ± 0.008 ^b–i^	1.091 ± 0.033 ^f–i^
Mean ± SD	82.46 ± 2.1	3.26 ± 1.84	25.33 ± 3.98	291 ± 124.1	0.65 ± 0.16	1.17 ± 0.36
CV (%)	2.55	56.29	15.72	42.65	25.28	31.17
Skewness	−0.81	0.55	0.79	0.88	0.27	0.44
Kurtosis	0.67	0.26	−0.01	0.99	0.26	−0.46

^1^ Data are presented as mean ± SD. Different lowercase letters in the same column indicate significant differences (*p* < 0.05). ^2^ L, lightness; a, red-green value; b, yellow-blue value. ^3^ TPC, total phenolic content; DPPH/ABTS, DPPH/ABTS radical scavenging activity.

**Table 2:** Composition of phenolic acids in *Cucurbita pepo* accessions ^1^.

Accessions	GA	PA	*p*-HA	VA	CaA	*p*-CA	SA	FA	TPA ^2^
S1	ND	ND	25.15 ± 0.52 ^op^	3.74 ± 0.15 ^hi^	Tr	2.77 ± 0.04 ^jk^	ND	8.78 ± 0.09 ^lmn^	40.45 ± 0.72 ^qr^
S2	ND	2.19 ± 0.3 ^o^	27.09 ± 0.43 ^no^	5.02 ± 0.2 ^gh^	Tr	7 ± 0.03 ^jk^	ND	4.79 ± 0.03 ^stu^	46.08 ± 0.26 ^pqr^
S3	ND	5.18 ± 0.14 ^i–m^	8.97 ± 0.09 ^q^	Tr	2.21 ± 0.06 ^bcd^	8.91 ± 0.19 ^jk^	ND	7.21 ± 0.07 ^opq^	32.49 ± 0.18 ^r^
S4	ND	13.54 ± 0.27 ^bc^	119.39 ± 0.98 ^f^	5.88 ± 0.27 ^g^	1.24 ± 0.09 ^g–j^	169.19 ± 0.4 ^f^	ND	65.33 ± 0.13 ^a^	374.57 ± 1.42 ^fg^
S5	ND	4.88 ± 0.27 ^i–n^	213.01 ± 6.8 ^d^	3.25 ± 0.05 ^ij^	Tr	180.77 ± 0.79 ^f^	8.78 ± 0.26 ^d^	ND	410.69 ± 7.01 ^e^
S6	ND	7.98 ± 0.11 ^efg^	45.71 ± 0.03 ^m^	3.47 ± 0.07 ^ij^	Tr	224.71 ± 0.37 ^e^	9.06 ± 0.32 ^d^	3.84 ± 0.07 ^uv^	294.77 ± 0.56 ^hi^
S7	ND	5.43 ± 0.44 ^i–l^	87.64 ± 1.05 ^hi^	7.28 ± 0.39 ^ef^	1.97 ± 0.09 ^def^	5.69 ± 0.02 ^jk^	ND	10.07 ± 0.18 ^lm^	118.07 ± 0.46 ^mn^
S8	ND	12.07 ± 0.6 ^cd^	79.64 ± 1.72 ^ijk^	5.61 ± 0.1 ^g^	1.52 ± 0.14 ^f–j^	5.09 ± 0.01 ^jk^	ND	8.49 ± 0.07 ^no^	112.42 ± 2.28 ^n^
S9	ND	12.72 ± 0.16 ^bcd^	27.52 ± 0.62 ^no^	8.61 ± 0.1 ^bcd^	Tr	498.2 ± 6.04 ^b^	24.89 ± 1.09 ^b^	ND	571.94 ± 6.45 ^b^
S10	ND	5.86 ± 0.38 ^h–l^	30.63 ± 0.34 ^no^	2.61 ± 0 ^ij^	1.55 ± 0.02 ^e–j^	2.42 ± 0.01 ^k^	ND	7.84 ± 0.03 ^nop^	50.92 ± 0.07 ^o–r^
S11	ND	8.77 ± 0.19 ^e^	14.94 ± 0.15 ^pq^	9 ± 0 ^bc^	1.08 ± 0 ^ij^	9.28 ± 0 ^jk^	ND	17.18 ± 0.05 ^gh^	60.25 ± 0.3 ^op^
S12	ND	3.96 ± 0.16 ^l–o^	14.91 ± 0.06 ^pq^	2.71 ± 0 ^ij^	1.54 ± 0.06 ^e–j^	8.9 ± 0.02 ^jk^	ND	4.11 ± 0.01 ^tu^	36.12 ± 0.02 ^r^
S13	ND	3.06 ± 0.14 ^no^	22.45 ± 0.09 ^op^	9.22 ± 0.52 ^bc^	1.69 ± 0.08 ^d–h^	2.72 ± 0.03 ^jk^	ND	6.71 ± 0.06 ^pqr^	45.84 ± 0.76 ^pqr^
S14	ND	4.42 ± 0.3 ^j–n^	153.17 ± 0.25 ^e^	2.41 ± 0 ^j^	2.12 ± 0.01 ^cde^	104.63 ± 1.51 ^g^	3.32 ± 0.17 ^e^	13.36 ± 0.24 ^jk^	283.43 ± 1.88 ^ij^
S15	ND	8.94 ± 0.52 ^e^	36.63 ± 0.98 ^mn^	3.2 ± 0.15 ^ij^	1.73 ± 0.04 ^d–h^	72.17 ± 0.06 ^h^	2.62 ± 0.02 ^e^	10.22 ± 0.01 ^l^	135.51 ± 1.64 ^lm^
S16	1.64 ± 0.05	13.37 ± 0.82 ^bc^	70.23 ± 0.43 ^kl^	3 ± 0.15 ^ij^	1.63 ± 0.34 ^d–i^	7.49 ± 0 ^jk^	ND	12.55 ± 0.01 ^k^	109.92 ± 0.61 ^n^
S17	ND	11.22 ± 0.25 ^d^	66.69 ± 0.28 ^l^	2.83 ± 0.07 ^ij^	4.03 ± 0.03 ^a^	44.61 ± 0.06 ^i^	ND	19.42 ± 0.2 ^f^	148.8 ± 0.34 ^l^
S18	ND	7.49 ± 0.93 ^e–h^	42.44 ± 1.26 ^m^	5.44 ± 0.22 ^g^	3.88 ± 0.08 ^a^	56.56 ± 0.06 ^i^	ND	31.2 ± 0.04 ^e^	147.02 ± 0.54 ^l^
S19	ND	8.28 ± 0.08 ^ef^	102.07 ± 1.2 ^g^	5.8 ± 0.29 ^g^	1.78 ± 0.06 ^d–g^	42.21 ± 0.19 ^i^	ND	64.52 ± 0.16 ^a^	224.67 ± 1.29 ^k^
S20	ND	12.91 ± 0.25 ^bcd^	61.64 ± 1.26 ^l^	9.22 ± 0.02 ^bc^	1.9 ± 0.17 ^def^	398.54 ± 3.99 ^c^	35.02 ± 0.72 ^a^	15.7 ± 0.7 ^hi^	534.94 ± 2.85 ^c^
S21	ND	14.3 ± 0.11 ^b^	93.61 ± 0 ^gh^	5.71 ± 0.15 ^g^	2.57 ± 0.06 ^bc^	761.88 ± 8.76 ^a^	ND	12.78 ± 0.19 ^jk^	890.85 ± 8.93 ^a^
S22	ND	12.04 ± 0.25 ^cd^	43.18 ± 0.09 ^m^	9.07 ± 0.02 ^bc^	3.75 ± 0.1 ^a^	372.62 ± 5.15 ^d^	13.11 ± 0.22 ^c^	17.24 ± 0.1 ^gh^	471.03 ± 5.24 ^d^
S23	ND	5.1 ± 0.38 ^i–m^	77.49 ± 1.29 ^ijk^	2.98 ± 0.07 ^ij^	1.15 ± 0.04 ^hij^	18.07 ± 0.62 ^j^	Tr	2.53 ± 0.24 ^v^	107.32 ± 1.25 ^n^
S24	ND	7.69 ± 0.19 ^e–h^	80.53 ± 0.03 ^ij^	9.84 ± 0.05 ^b^	Tr	180.02 ± 2.43 ^f^	8.21 ± 0.46 ^d^	14.32 ± 0.54 ^ij^	300.6 ± 2.46 ^hi^
S25	ND	21.65 ± 0.44 ^a^	71.36 ± 0.22 ^jkl^	3.42 ± 0.02 ^ij^	1.22 ± 0.02 ^g–j^	367.17 ± 7.57 ^d^	ND	5.62 ± 0.49 ^rst^	470.45 ± 8.66 ^d^
S26	ND	8.64 ± 0.33 ^ef^	29.37 ± 0.25 ^no^	6.03 ± 0.22 ^fg^	1.26 ± 0.04 ^g–j^	5.32 ± 0.03 ^jk^	ND	6.47 ± 0.2 ^pqr^	57.08 ± 0.09 ^opq^
S27	ND	4.01 ± 0.05 ^k–o^	42.51 ± 0.58 ^m^	9.22 ± 0.42 ^bc^	Tr	4.32 ± 0.08 ^jk^	ND	5.8 ± 0.26 ^qrs^	65.85 ± 0.22 ^o^
S28	ND	9.13 ± 0.05 ^e^	341.98 ± 1.51 ^a^	5.66 ± 0.1 ^g^	1.04 ± 0.05 ^j^	13.59 ± 0.08 ^jk^	ND	16.06 ± 0.75 ^h^	387.47 ± 2.43 ^f^
S29	ND	3.33 ± 0.08 ^mno^	45.61 ± 0.62 ^m^	8.24 ± 0.12 ^cde^	Tr	3.39 ± 0.01 ^jk^	ND	8.61 ± 0.33 ^mno^	69.85 ± 0.26 ^o^
S30	ND	6.02 ± 0.22 ^g–k^	239.59 ± 2.37 ^c^	6.15 ± 0.39 ^fg^	2.07 ± 0.05 ^c–f^	9.39 ± 0.04 ^jk^	ND	44.58 ± 0.09 ^c^	307.8 ± 2.72 ^h^
S31	ND	6.71 ± 0.46 ^f–i^	281.16 ± 3.88 ^b^	11.21 ± 0.05 ^a^	2.78 ± 0.09 ^b^	14.57 ± 0.03 ^jk^	ND	55.37 ± 0.07 ^b^	371.79 ± 3.38 ^fg^
S32	ND	6.08 ± 0.27 ^g–j^	234.76 ± 3.45 ^c^	6.03 ± 0.32 ^fg^	Tr	6.12 ± 0.04 ^jk^	ND	18.44 ± 0.22 ^fg^	271.43 ± 3.14 ^j^
S33	ND	14.63 ± 0.16 ^b^	280.7 ± 1.26 ^b^	7.7 ± 0.47 ^de^	1.71 ± 0.09 ^d–h^	13.12 ± 0.03 ^jk^	ND	38.56 ± 0.12 ^d^	356.41 ± 1.51 ^g^
Median	0.00	7.69	66.69	5.71	1.54	13.59	0.00	10.22	148.80
CV (%)	565.69	54.21	93.56	46.98	79.01	161.19	239.79	101.93	82.70
Skewness	5.74	0.76	1.46	0.10	0.49	2.16	3.07	1.77	1.22
Kurtosis	33.00	0.89	1.11	−0.82	−0.03	4.74	9.83	2.35	1.80

^1^ ND, not detected; Tr, trace (<1 μg/g). “ND” and “Tr” were recorded as 0 for calculation purposes. Different lowercase letters in the same column indicate significant differences (*p* < 0.05). ^2^ TPA, total phenolic acid content. Other abbreviations are shown in Figure 1.

## Data Availability

The original contributions presented in this study are included in the article/Supplementary Materials. Further inquiries can be directed to the corresponding authors.

## Supplementary Materials

The following supporting information can be downloaded at https://www.mdpi.com/article/10.3390/foods15122226/s1, Table S1: Regression equations, limits of detection (LOD), limits of quantification (LOQ), and recovery rates for analysis of the phenolic acids; Table S2: Loading matrix in the principal component analysis.

## Abbreviations

The following abbreviations are used in this manuscript:

CaA	Caffeic acid
FA	Ferulic acid
GA	Gallic acid
HPLC	High-performance liquid chromatography
PA	Protocatechuic acid
*p*-CA	*p*-Coumaric acid
*p*-HA	*p*-Hydroxybenzoic acid
SA	Sinapic acid
TPC	Total phenolic content
TPA	Total phenolic acid content (sum of all quantified phenolic acid fractions)
VA	Vanillic acid
